# Evaluation of the impact of disease prevention measures: a methodological note on defining incidence rates

**DOI:** 10.1186/s12874-017-0350-4

**Published:** 2017-04-21

**Authors:** Yin-Bun Cheung, Ying Xu, Matthew Cairns, Paul Milligan

**Affiliations:** 1 Center for Quantitative Medicine, Duke-NUS Medical School, Level 6, Academia, 20 College Road, Singapore, 169856 Singapore; 2 Tampere Center for Child Health Research, University of Tampere and Tampere University Hospital, ARVO Building, FIN-33014 Tampere, Finland; 3 Department of Infectious Disease Epidemiology, London School of Hygiene and Tropical Medicine, Keppel Street, WC1E 7HT London, UK

**Keywords:** Incidence rate, Prevention trials, Protective efficacy, Recurrent events

## Abstract

**Background:**

In studies of recurrent events, it is common to consider a person who has suffered a disease episode and received curative treatment to be not at risk of suffering a new episode for a duration of time. It is a common practice to deduct this duration from the person’s observation time in the statistical analysis of the incidence data.

**Methods:**

We examined the concepts of incidence and protective efficacy from a real life point of view. We developed simple formulae to show the relationship between the incidence rate and protective efficacy between analyses with and without deducting the curative treatment time from the observation time. We used a malaria chemoprevention and a malaria vaccine study, both previously published, to illustrate the differences.

**Results:**

Applying the formulae we derived to a range of disease incidence that covered the two case studies, we demonstrated the divergence of the two sets of estimates when incidence rate is approximately 1 per person-year or higher. In the malaria chemoprevention study, incidence was 5.40 per person-year after the deduction of curative treatment time from observation time but 4.48 per person-year without the deduction. The chemoprevention offered 56.6 and 50.7% protection calculated with and without the deduction, respectively. In the malaria vaccine study, where disease incidence was much lower than one, the results between the two ways of analysis were similar. For answering real life questions about disease burden in the population in a calendar year and the reduction that may be achieved if an intervention is implemented, the definition without deduction of curative treatment time should be used.

**Conclusions:**

The practice of deducting curative treatment time from observation time is not wrong, but it is not always the best approach. Investigators should consider the appropriateness of the two analytic procedures in relation to the specific research aims and the intended use of the results.

**Electronic supplementary material:**

The online version of this article (doi:10.1186/s12874-017-0350-4) contains supplementary material, which is available to authorized users.

## Background

Incidence rate is defined as the number of events divided by the duration of person-time [1–4]. In regression analysis of disease incidence to evaluate intervention effects, e.g. by Poisson regression or Negative Binomial regression, the duration of person-time is used as an off-set variable and the way the duration is defined affects the estimated incidence and incidence rate ratio [5]. In time-to-event analysis, the duration determines who is to be included in the risk set for evaluation. While the case definition of a disease episode (in the numerator) and the technical aspects of statistical analysis methods are often detailed in study reports, the issue of defining the denominator of incidence rates has received less attention than it should.

In prevention trials and epidemiological studies of disease incidence, the numerator of an incidence rate is often defined by the occurrence of a set of signs and symptoms and objective measurements plus the fact of presentation to a health care facility for treatment. For example, in a malaria vaccine trial, malaria was defined as temperature ≥ 37.5 °C or self-report of fever in the last 24 h, malaria parasitemia ≥ 2500 per μL, and presentation to a health care facility [6, 7].

Definitions of the denominator of an incidence rate vary subtly. Table 1 shows the denominators given in some popular references. Some of them used the phrase “at risk” in the definition, but some did not. Porta’s Dictionary of Epidemiology described a “person-time incidence rate” that used “number of person-time units at risk” in the denominator and another definition that does not involve time at all (the latter is not shown in table).

**Table 1:** The denominators of incidence rate as defined in some references

Source	Denominator
Hennekens and Buring ([1], p.57)	“total person-time of observation”
Smith and Morrow ([2], p.306)	“person-time-at-risk”
Greenland and Rothman ([3], p.34)	“time spent in population”
Porta ([4], online version])	“number of person-time units at risk”

It is a common practice to consider some duration of time after each episode of disease as a time period that the person is not “at risk” of the disease. For example, in studies of malaria vaccines and chemoprevention, a person who is known to have clinical malaria is given curative treatment. Antimalarials persist at therapeutic levels for a variable period depending on the pharmacokinetics of the specific drugs [6–8]. It is often assumed that malaria symptoms occurring “early” (defined variably) after initiation of curative treatment are the results of the initial infection and not the result of a new infection. The early occurrences of these symptoms are not counted as disease episodes in the numerator. A variable amount of time is then deducted from the person-time in the denominator of the incidence rate, usually 7 to 28 days depending on the specific malaria drugs used, apparently with the aim of excluding the period during which the person is supposed to be not at risk. The person-time is then said to be “adjusted for anti-malaria drug use” [6–9]. Similar practices can be found in other therapeutic areas, such as the management of pulmonary exacerbations in cystic fibrosis [10, 11] and prevention of pneumonia [12].

We define “observation time” as the total duration of time a subject is under observation, i.e. from entry to exit from study minus temporary absence from observation (if any), e.g. due to migration. Deducting curative treatment time from observation time gives “time at risk”, a denominator that is smaller than that based on observation time. For brevity, we refer to the definitions of incidence rate with and without the deduction of treatment time as “time at risk” definition and “observation time” definition. Not much discussion has been dedicated to the appropriateness of this practice of deducting the curative treatment time. Some statistics textbooks briefly mention this issue and adopt the time at risk definition [10, 11].

We maintain that not counting the early occurrences of symptoms in the numerator does not necessitate the deduction of the treatment time from the denominator, and that the practice of the deduction does not answer the questions policy makers and health programme managers seek to answer. From a real life point of view, whether in terms of efficacy or effectiveness, it is useful to know the disease burden in terms of how many disease episodes there are per calendar year in a population and how many episodes may be prevented by the introduction of an intervention per calendar year. In this context, time is the observation time a health policy or program is under evaluation. The practice of subtracting a period of curative treatment time from the actual observation time does not answer these questions. As an analogy, we find the “observation time” definition similar to intention-to-treat analysis, while the “time at risk” definition similar to per-protocol analysis [13, 14]. Intention-to-treat analysis aims to obtain the fairest estimate of the intervention benefit that would be realized in practice, and therefore does not exclude participants who deviate from the protocol in ways which could occur in real life situations, such as taking other medications or non-adherence to the intervention. In studies of preventive interventions, curative treatment of disease temporarily reduces the risk of a future disease episode. This is a feature of routine health care and so excluding the curative treatment time from the analysis is not compatible with research questions concerning real life situations.

## Methods

### Relationship between estimates

For the purpose of illustration, consider a 2-arm, randomized controlled trial of a preventive intervention versus placebo control.

#### Disease incidence

Let the observation time for subject i in group j be defined as T_ij_ = min(τ, C_ij_), where τ is the maximum follow-up duration fixed by study design and C_ij_ is the non-informative right-censored total duration of time subject i in group j is under observation, i.e. the time from entry to exit from study minus duration of temporary absence from observation (if any), e.g. due to migration, and j = 0 for control group and 1 for intervention group. Let T_j_ = ∑_i_T_ij_ be the total amount of observation time summed across all subjects in group j. Let D_ijk_ be the curative treatment time for subject i in group j after the k-th event. To simplify notations and focus on the concepts instead of technicalities, we assume in this article that D_i1k_ = D_i0k_ = D. However, this assumption is not central to our argument and we will revisit this in the Discussion section. The practice of deducting curative treatment time from observation time assumes that there is no new episode of disease during the treatment period, D; any symptom occurrence observed in this period are considered relapses and not counted in the numerator.

Let E_ij_ denote the number of episodes for subject i in group j. Let E_j_ = ∑_i_E_ij_ be the total number of episodes observed in group j. Let I_j_ be the incidence rate defined as total number of episodes divided by T_j_, without deduction of the treatment time after each episode, in group j. That is, the “observation time” definition of incidence rate is:

$$ {\mathrm{I}}_{\mathrm{j}}={\mathrm{E}}_{\mathrm{j}}/{\mathrm{T}}_{\mathrm{j}} $$

If the curative treatment time is deducted from the observation time after each event, the “time at risk” definition of incidence rate obtained is approximately:

$$ {\mathrm{I}}_{\mathrm{j}}^{*}={\mathrm{E}}_{\mathrm{j}}/\left({\mathrm{T}}_{\mathrm{j}}-\left({\displaystyle {\sum}_{\mathrm{i}}}{\mathrm{E}}_{\mathrm{i}\mathrm{j}}\mathrm{D}\right)\right) $$

or equivalently

1 $$ {\mathrm{I}}_{\mathrm{j}}^{*}={\mathrm{E}}_{\mathrm{j}}/\left({\mathrm{T}}_{\mathrm{j}}-{\mathrm{E}}_{\mathrm{j}}\mathrm{D}\right) $$

Equation 1 is approximate because if the last episode occurred within duration D from the end of the observation time, the deduction for the last episode would be smaller than D. In the Additional file 1 we show that an improved estimate of the deduction of treatment time after the last event in subject i of group j is

$$ \frac{{\mathrm{T}}_{\mathrm{ij}}-\left({\mathrm{E}}_{\mathrm{ij}}-1\right)\mathrm{D}}{{\mathrm{E}}_{\mathrm{ij}}+1}\left\{1-{\left(1-\frac{\mathrm{D}}{{\mathrm{T}}_{\mathrm{ij}}-\left({\mathrm{E}}_{\mathrm{ij}}-1\right)\mathrm{D}}\right)}^{{\mathrm{E}}_{\mathrm{ij}}+1}\right\} $$

Therefore, the “time at risk” definition of incidence rate is

2 $$ {\mathrm{I}}_{\mathrm{j}}^{*}=\frac{{\mathrm{E}}_{\mathrm{j}}}{{\mathrm{T}}_{\mathrm{j}}-{\displaystyle {\sum}_{\mathrm{i}}}\left[\left({\mathrm{E}}_{\mathrm{i}\mathrm{j}}-1\right)\mathrm{D}+\frac{{\mathrm{T}}_{\mathrm{i}\mathrm{j}}-\left({\mathrm{E}}_{\mathrm{i}\mathrm{j}}-1\right)\mathrm{D}}{{\mathrm{E}}_{\mathrm{i}\mathrm{j}}+1}\left\{1-{\left(1-\frac{\mathrm{D}}{{\mathrm{T}}_{\mathrm{i}\mathrm{j}}-\left({\mathrm{E}}_{\mathrm{i}\mathrm{j}}-1\right)\mathrm{D}}\right)}^{{\mathrm{E}}_{\mathrm{i}\mathrm{j}}+1}\right\}\right]} $$

**Fig. 1:**
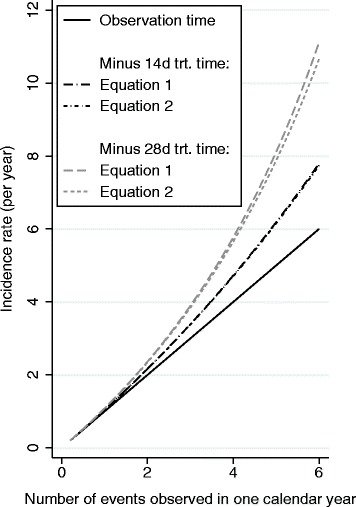
Incidence rates defined with and without the deduction of curative treatment time

As will be seen in Fig. 1, for realistic values of T_j_ and D in public health studies, Eq. 1 gives very good approximation to Eq. 2. We focus on Eq 1 in subsequent discussion for simplicity.

If D = 0, I_j_^*^ = I_j_. Otherwise, I_j_^*^ > I_j_ due to the smaller denominator in I_j_^*^. In other words, the incidence rate according to the “time at risk” definition is larger than that of the “observation time” definition.

Furthermore, from (1), it can be shown that E_j_ = (I_j_^*^T_j_)/(1 + I_j_^*^D). Therefore,

3 $$ {\mathrm{I}}_{\mathrm{j}}={\mathrm{I}}_{\mathrm{j}}^{*}/\left(1+{\mathrm{I}}_{\mathrm{j}}^{*}\mathrm{D}\right) $$

From Eq. 3, it can be seen again that I_j_ is smaller than I_j_^*^ unless D equals zero.

#### Protective efficacy

Protective efficacy (PE) is defined as 1 minus incidence rate ratio [15, 16]. We use the phrase here statistically to mean 1 – incidence rate ratio, without making a distinction between the contexts of efficacy or effectiveness studies.

Let R = I_1_/I_0_ and R* = I_1_^*^/I_0_^*^, i.e. the incidence rate ratio comparing the intervention to control group based on the “observation time” and “time at risk” definitions, respectively. By substituting I_1_^*^ = R*I_0_^*^ into Eq. 3:

4 $$ {\mathrm{I}}_1=\left({\mathrm{R}}^{*}{\mathrm{I}}_0^{*}\right)/\left(1+{\mathrm{R}}^{*}{\mathrm{I}}_0^{*}\mathrm{D}\right) $$

Let PE = 1 − R and PE* = 1 − R*.

5 $$ \begin{array}{rcl}\mathrm{PE}& =& 1-\left[\left({\mathrm{R}}^{*}{\mathrm{I}}_0^{*}\right)/\left(1+{\mathrm{R}}^{*}{\mathrm{I}}_0^{*}\mathrm{D}\right)\right]/\left[{\mathrm{I}}_0^{*}/\left(1+{\mathrm{I}}_0^{*}\mathrm{D}\right)\right]\\ {}\ & =& {\mathrm{PE}}^{*}/\left(1+{\mathrm{I}}_1^{*}\mathrm{D}\right)\end{array} $$

As such, PE* > PE unless D equals zero. The equality in Eq. 5 also holds if PE = 0. As such, for the purpose of testing a null hypothesis of no intervention effect, using PE or PE^*^ does not matter. However, the “time at risk” definition tends to give a stronger estimate of protective efficacy than the “observation time” definition unless there is no intervention effect at all.

## Results

Figure 1 illustrates the discrepancy between the incidence rates defined with and without the deduction of curative treatment time. This figure covers the range of incidence rates that includes the two case studies we will discuss. The x-axis is the number of episodes observed in one calendar year. The incidence rates based on “observation time” (solid line) formed a 45° line to the x-axis. In contrast, the incidence rates based on “time at risk” after deduction of 14 days or 28 days of treatment time (typical in malaria studies) following each episode had an accelerating slope. They began to depart visibly from the rates based on the “observation time” definition when incidence was about 1 per year. The gap expanded as incidence increased. Furthermore, the approximate (Eq. 1) and precise (Eq. 2) versions of the incidence rate estimates based on the “time at risk” definition were practically identical when 14 days were deducted. There was visible but minor difference between the approximate and precise versions when 28 days were deducted. This demonstrates the usefulness of Eq. 1.

**Fig. 2:**
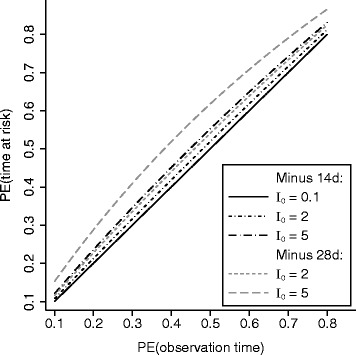
Differences in estimates of protective efficacy with and without the deduction of curative treatment time - PE(time at risk) and PE(observation time), respectively - depend on background incidence

Figure 2 contrasts the two definitions of protective efficacy in the cases of incidence in the control group being 0.1, 2, and 5 episodes in one calendar year of observation time, which roughly correspond to the range in the two case studies below. The lines for deduction of 14 and 28 days were practically identical at low incidence (0.1 per year), and hence only the line for deduction of 14 days was shown for 0.1 per year. For low incidence (0.1 per year), PE^*^ and PE almost exactly formed a 45° line, indicating strong agreement. As incidence became higher, PE^*^ became larger than PE. The deduction of 28 days generated bigger difference between PE^*^ and PE than the deduction of 14 days did.

We use two different malaria prevention studies to illustrate.

### Study 1. Chemoprevention of malaria in Ugandan children

Three hundred and ninety three infants at 6 months of age were randomized to no chemoprevention, monthly sulfadoxine-pyrimethamine (SP), daily trimethoprim-sulfamethoxazole (TS), or monthly dihydroartemisinin-piperaquine (DP) [8]. Chemoprevention ended at the age of 24 months. Passive surveillance of malaria incidence was conducted. A malaria diagnosis was defined as temperature at least 38.0° Celsius or history of fever in the previous 24 h and a positive thick blood smear. Malaria was treated according to local clinical guidelines using either artemether-lumefantrine or quinine. The study deducted 14 days after each malaria attack from the denominator. Negative binomial regression was used to analyze the incidence data.

Pooling four groups, the overall incidence was 5.404 per person-year at risk (PYAR) after the deduction of curative treatment time from the observation time (Table 2). Using Eq. 3, the incidence based on observation time would be 4.477 per year. The former estimate of incidence was about 1 episode per time unit higher than the latter. DP was found to offer the highest protection: based on the “time at risk” definition this was 1–3.017/6.953 = 56.6%, but using Eq. 5, the PE based on observation time was lower: 0.566/[1 + 3.017 × (14/365.25)] = 0.507, or 50.7%.

**Table 2:** Chemoprevention of malaria in Ugandan children, 2010–2013

Trial arm	Sample size	Number of events	Person-year at risk^a^	Event/PYAR	Event/observation time^b^
Control	98	760	109.3	6.953	5.490
SP	98	725	107.8	6.725	5.347
TS	99	609	116.8	5.214	4.346
DP	98	366	121.3	3.017	2.704
Overall	393	2460	455.2	5.404	4.477

^a^Observation time minus malaria curative treatment time

^b^Estimated using Eq. 3

### Study 2. RTS,S malaria vaccine in Mozambican children

One thousand four hundred ninety three children aged 1 to 4 years were recruited and randomized to receive either control vaccines or RTS,S malaria vaccine [6, 7]. The surveillance period started at 14 days after the third dose of vaccine, which was 2.5 months post-enrollment. Surveillance continued to 21.0 months post-enrollment. Malaria was defined as temperature ≥ 37.5 °C or reporting fever in the last 24 h and malaria parasitemia ≥ 2500 per μL. After exclusion of 3 children from per-protocol analysis, 1490 were included in the main analysis. Several drugs were used in the treatment of malaria. In the analysis of multiple episode data, a child was considered not at risk for 28 days after the onset of the previous event due to treatment [6, 7].

The overall disease incidence was 0.351 events per PYAR according to the “time at risk” definition (Table 3). Using Eq. 3 to estimate the incidence based on observation time, the incidence was 0.342. The PE based on the definition with deduction of curative treatment time was 1–0.309/0.395 = 21.9%. Using Eq. 5, based on observation time, a similar PE of 21.4% was obtained. The PE in the trial report was adjusted for age, bednet use, and other covariates, and therefore was somewhat different [7].

**Table 3:** RTS,S Malaria vaccine in Mozambican children, 2003–2005

Trial arm	Sample size	Number of event	Person-year at risk^a^	Events/PYAR	Events/observation time^b^
Control	745	384	972.1	0.395	0.383
RTS,S	745	310	1004.5	0.309	0.301
Overall	1490	694	1976.6	0.351	0.342

^a^Observation time minus malaria curative treatment time

^b^Estimated using Eq. 3

## Discussion

Epidemiological studies and clinical trials typically are meticulous about case definitions and statistical analysis techniques. Less attention has been given to the denominator of an incidence rate. The definitions of the numerator and denominator are two distinct matters. We considered the denominator while taking the numerator definition as given. While some textbooks and references do use the phrase “at risk” in the definition, it is not clear what exactly “at risk” means. Some investigations, such as the malaria prevention trials we discussed, consider a person not at risk while they were receiving curative treatments because the curative treatments were supposed to temporarily make the person non-susceptible to the target disease. This definition appears to interpret “at risk” to mean “biologically susceptible”. However, it is possible to interpret “at risk” as being under observation. For example, in the RTS,S vaccine trial in Mozambique, children were also considered not at risk if they were absent from the study area for at least 2 weeks [6, 7]. Perhaps the children were biologically susceptible to malaria during their absence, but they were not under observation by the study team and this was the ground for excluding the duration of time from the statistical analysis. Interpreting this way, time at risk is equivalent to observation time.

In our opinion, both the definitions of incidence rate and protective efficacy with or without the deduction of treatment time are legitimate. However, they concern different research questions. We maintain that, from a real life and public health point of view, the observation time definition tends to be more appropriate, because a policy maker or programme manager is likely more concerned about what would occur in the community in a calendar year if an intervention is or is not implemented. In this context, time refers to observation time in the real life situation. The choice between the two denominators may or may not make a practically important difference. This depends on the disease incidence in the population. When disease incidence is high, from a real life perspective, disease incidence and protective efficacy can be substantially over-estimated by the common practice. When disease incidence is low, the difference may not be noticeable. These issues should be considered in study designs and in planning analyses.

It is good that many studies do present enough information that allows reconstruction of the incidence rate from one definition to another. The equations we presented should facilitate this. The equations do not directly provide confidence intervals because that would require individual level data instead of published estimates. But they can be applied to both the point estimates and the lower and upper limits of their confidence intervals in published reports to obtain the reconstructed point and interval estimates. However, this conversion based on published information is only possible for crude incidence. It is not possible for covariate (or random effects) adjusted analysis as the individual level data is typically not available to readers. As such, it is important for investigators to consider the appropriateness of the two analysis approaches in relation to the specific research context and accordingly provide the adjusted analysis to readers if needed. It is quite common that clinical trials present both intention-to-treat analysis and per protocol analysis results. Similarly, investigators may consider presenting both versions of incidence rate and protective efficacy. They are not mutually exclusive. In the derivation of the conversion formulae in the Methods we made an assumption of constant curative treatment time. The assumption was made primarily for brevity of exposition. It is not central to the conclusions we made. Similar to the paucity of discussion on the present topic, there is also a paucity of information about the constant curative treatment time assumption. It will be helpful if studies on disease prevention also provide information on curative treatments. The accuracy of the conversion formulae developed in the Methods section would be affected if the distribution of curative treatment time is neither constant nor random. We can imagine the possibility that curative treatment may depend on number of previous disease episodes or disease severity, possibly leading to unequal distribution of curative treatment times across intervention groups. If that occurs, the results based on the “time at risk” definition would be difficult to interpret, regardless of using the conversion formulae or not. In our opinion, that would strengthen the motivation for using the “observation time” definition.

If the “time at risk” definition is chosen, it is important to present the operational details, which have not been always clear in the literature. For example, it is quite common in vaccine studies to start observation when a participant is enrolled, but the analysis time starts only when the series of vaccination (e.g. three doses) is completed. If a disease episode is observed before the date of completion of vaccine series but the curative treatment time extends beyond this date, we believe the logic behind the choice of the “time at risk” definition should mandate deduction of the part of curative treatment time that is after this date. Insufficient description hinders understanding and reproducibility. An alternative approach, which avoids making specific assumptions about at-risk status during curative treatment, is to use a time-varying covariate [10, 11] for the preventive intervention variable so that the protective efficacy may change according to curative treatment history.

## Conclusions

The practice of deducting treatment time from observation time is not wrong, but it is not always the best approach. It is important for investigators to consider the appropriateness of the two forms of analysis in relation to the specific research aims and the intended use of the results.

## Additional file

Additional file 1: “Time not at risk” and its approximation. Derivation of the equations for approximating time not at risk. (DOCX 33 kb)

## Abbreviations

DP: Dihydroartemisinin-piperaquine
PE: Protective efficacy
PYAR: Person-year at risk
SP: Sulfadoxine-pyrimethamine
TS: Trimethoprim-sulfamethoxazole
