# Supplementary material for: Evaluation of the impact of disease prevention measures: a methodological note on defining incidence rates
Source: BMC Med Res Methodol. 2017 Apr 21;17:72. doi: 10.1186/s12874-017-0350-4 (PMC5401565; doi:10.1186/s12874-017-0350-4)
Supplement: Additional file 1: — “Time not at risk” and its approximation. Derivation of the equations for approximating time not at risk. (DOCX 33 kb) [file 12874_2017_350_MOESM1_ESM.docx]

Additional file 1

“Time not at risk” and its approximation

Let $E_{\mathrm{ij}}$ be the total number of outcome events observed in subject i in group j over a total amount of observation time $T_{\mathrm{ij}}$. Let the treatment time after the onset of an event be a constant D(“time not at risk”). The practice of deducting treatment time from observation time assumes that there is no new event during this period of time, D; any events observed in this period are considered relapses and hence not counted in the numerator. Let $T_{\mathrm{ij}}^{*}$denote the total amount of at risk time obtained after deducting the total treatment time after each event from the total observation time $T_{\mathrm{ij}}$. Define $E_{j}=\sum_{i} E_{\mathrm{ij}}$, $T_{j}=\sum_{i} T_{\mathrm{ij}}$ and $T_{j}^{*}=\sum_{i} T_{\mathrm{ij}}^{*}$. Assume that by study design, D is much smaller than $T_{j}$ ($D\ll T_{j}$) such that for all subjects, $T_{\mathrm{ij}}>E_{\mathrm{ij}}\times D$.

It is easy to see that $T_{\mathrm{ij}}$ and $T_{\mathrm{ij}}^{*}$ satisfies the following equation

${\bar{E}[T}_{\mathrm{ij}}-T_{\mathrm{ij}}^{*}]=\left( E_{\mathrm{ij}}-1 \right)\times D+\bar{E}[N.A.R.\left( E_{\mathrm{ij}} \right)]$ (A.1)

where the first term on the right hand side captures the total treatment time associated with the first $(E_{\mathrm{ij}}-1)$ events, and the second term $\bar{E}[N.A.R.(E_{\mathrm{ij}})]$ denotes the expected treatment time to be deducted from the denominator after the $E_{\mathrm{ij}}$th event. Here the notation $\bar{E}\left[ \cdot\right]$ represents a conditional expectation that will be detailed in the following part. And we will establish an explicit expression for $\bar{E}[N.A.R.(E_{\mathrm{ij}})]$.

We assume that the disease incidence rate remains constant over the follow-up period. This is a simplification as diseases may show seasonality. If truly the follow-up ends in a high season (as compared to a low season), it is more likely that the last event may occur near the end of the follow-up period and therefore the treatment time to be deducted according to the “time at risk” definition may be smaller than D and equation 1 become less accurate. However, it is uncommon to design a trial to end in a high season. A logical study design should start and end in a low season such that, given the same follow-up time, the number of observed events is maximized. In this design, the last event is unlikely to occur near the end of the follow-up period and therefore the treatment time to be deducted is close to D and equation 1 is accurate. The simplifying assumption here is a middle-of-the-road scenario. For the time being, we ignore the gap D between event times. Then given that $E_{\mathrm{ij}}$events are observed over a total amount of observation time $T_{\mathrm{ij}}$, the $E_{\mathrm{ij}}$event times follow a uniform distribution, over the interval [0, $T_{\mathrm{ij}}$]. This entails the following probability density function (p.d.f.) for the $E_{\mathrm{ij}}$ ordered event times $t_{\left( i,1 \right)},t_{\left( i,2 \right)}, \ldots,t_{\left( i,E_{\mathrm{ij}}-1 \right)}$ and$t_{\left( {i,E}_{\mathrm{ij}} \right)}$, conditional on $E_{\mathrm{ij}}$ and $T_{\mathrm{ij}}$:

$f\left( t_{\left( i,1 \right)},..., t_{\left( {i,E}_{\mathrm{ij}} \right)}| E_{\mathrm{ij}}, T_{\mathrm{ij}} \right)=E_{\mathrm{ij}}!\left( \frac{1}{T_{\mathrm{ij}}} \right)^{E_{\mathrm{ij}}}.$ (A.2)

Let $Z_{i,1}=t_{\left( i,2 \right)}-t_{\left( i,1 \right)},$ $Z_{i,2}=t_{\left( i,3 \right)}-t_{\left( i,2 \right)}$, …., $Z_{{i,E}_{\mathrm{ij}}-1}=t_{\left( {i,E}_{\mathrm{ij}} \right)}-t_{\left( i, E_{\mathrm{ij}}-1 \right)}$, $Z_{{i,E}_{\mathrm{ij}}}=T_{\mathrm{ij}}-t_{\left( {i,E}_{\mathrm{ij}} \right)}$, where $Z_{i,1}$ to $Z_{{i,E}_{\mathrm{ij}}-1}$ denote the gap time between every two successive events, and $Z_{{i,E}_{\mathrm{ij}}}$ denotes the elapsed time from the last event till the end of follow-up. Based on (A.2), it follows immediately that the joint p.d.f. of $Z_{i,1},\ldots, Z_{{i,E}_{\mathrm{ij}}}$, conditional on $E_{\mathrm{ij}}$ and $T_{\mathrm{ij}}$, is

$f\left( Z_{i,1},\ldots, Z_{{i,E}_{\mathrm{ij}}}| E_{\mathrm{ij}}, T_{\mathrm{ij}} \right)=E_{\mathrm{ij}}!\left( \frac{1}{T_{\mathrm{ij}}} \right)^{E_{\mathrm{ij}}}$. (A.3)

Now, given the gap time between every two successive events is at least D and suppose the gap time between the last event and the end of follow-up is $z$, the corresponding joint probability, conditional on $E_{\mathrm{ij}}$ and $T_{\mathrm{ij}}$, is

$\Pr\left( Z_{i,1}\geq D,\ldots, Z_{{i,E}_{\mathrm{ij}}-1}\geq D, Z_{{i,E}_{\mathrm{ij}}}=z | E_{\mathrm{ij}}, T_{\mathrm{ij}} \right)$

$=\int_{D}^{T_{\mathrm{ij}}-z-\left( E_{\mathrm{ij}}-2 \right)D} \ldots{\int_{D}^{T_{\mathrm{ij}}-z-\left( E_{\mathrm{ij}}-2 \right)D} E_{\mathrm{ij}}!\left( \frac{1}{T_{\mathrm{ij}}} \right)^{E_{\mathrm{ij}}}}dZ_{i,1}\ldots dZ_{{i,E}_{\mathrm{ij}}-1}$

$=E_{\mathrm{ij}}!\left( \frac{1}{T_{\mathrm{ij}}} \right)^{E_{\mathrm{ij}}}\left( T_{\mathrm{ij}}-z-\left( E_{\mathrm{ij}}-1 \right)D \right)^{E_{\mathrm{ij}}-1}.$ (A.4)

Furthermore,

$$\Pr\left( Z_{i,1}\geq D,\ldots, Z_{{i,E}_{\mathrm{ij}}-1}\geq D | E_{\mathrm{ij}}, T_{\mathrm{ij}} \right)=\int_{0}^{T_{\mathrm{ij}}-\left( E_{\mathrm{ij}}-1 \right)D} E_{\mathrm{ij}}!\left( \frac{1}{T_{\mathrm{ij}}} \right)^{E_{\mathrm{ij}}}\left( T_{\mathrm{ij}}-z-\left( E_{\mathrm{ij}}-1 \right)D \right)^{E_{\mathrm{ij}}-1}dz$$

${=(E_{\mathrm{ij}}-1)!\left( \frac{1}{{T_{\mathrm{ij}}}} \right)}^{E_{\mathrm{ij}}}\left( T_{\mathrm{ij}}-\left( E_{\mathrm{ij}}-1 \right)D \right)^{E_{\mathrm{ij}}}$ . (A.5)

Therefore, the probability of $Z_{{i,E}_{\mathrm{ij}}}=z$, conditional on $E_{\mathrm{ij}}, T_{\mathrm{ij}}$ and all gap times corresponding to the first ($E_{\mathrm{ij}}-1$) events being at least D, is

$\Pr\left( Z_{{i,E}_{\mathrm{ij}}}=z | Z_{i,1}\geq D,\ldots, Z_{{i,E}_{\mathrm{ij}}-1}\geq D, E_{\mathrm{ij}}, T_{\mathrm{ij}} \right)=\frac{E_{\mathrm{ij}}\left( T_{\mathrm{ij}}-z-\left( E_{\mathrm{ij}}-1 \right)D \right)^{E_{\mathrm{ij}}-1}}{\left( T_{\mathrm{ij}}-\left( E_{\mathrm{ij}}-1 \right)D \right)^{E_{\mathrm{ij}}}}$ (A.6)

for 0$<z\leq T_{\mathrm{ij}}-\left( E_{\mathrm{ij}}-1 \right)D$.

Based on equation (A.6), we can calculate that the conditional expectation of $N.A.R\left( E_{\mathrm{ij}} \right)$, given $E_{\mathrm{ij}}, T_{\mathrm{ij}}$ and $Z_{i,1}\geq D,\ldots, Z_{{i,E}_{\mathrm{ij}}-1}\geq D$, is

$$\bar{E}\left[ N.A.R.\left( E_{\mathrm{ij}} \right) \right]=E\left[ N.A.R\left( E_{\mathrm{ij}} \right) | Z_{i,1}\geq D,\ldots, Z_{{i,E}_{\mathrm{ij}}-1}\geq D,E_{\mathrm{ij}}, T_{\mathrm{ij}} \right]=D\times\Pr\left( Z_{{i,E}_{\mathrm{ij}}}\geq D | Z_{i,1}\geq D,\ldots, Z_{{i,E}_{\mathrm{ij}}-1}\geq D,E_{\mathrm{ij}}, T_{\mathrm{ij}} \right)$$

$+\int_{0}^{D} \mathrm{zPr}\left( Z_{{i,E}_{\mathrm{ij}}}=z | Z_{i,1}\geq D,\ldots, Z_{{i,E}_{\mathrm{ij}}-1}\geq D, E_{\mathrm{ij}}, T_{\mathrm{ij}} \right)\mathrm{dz}$

$=D\times\int_{D}^{T_{\mathrm{ij}}-\left( E_{\mathrm{ij}}-1 \right)D} \frac{E_{\mathrm{ij}}\left( T_{\mathrm{ij}}-z-\left( E_{\mathrm{ij}}-1 \right)D \right)^{E_{\mathrm{ij}}-1}}{\left( T_{\mathrm{ij}}-\left( E_{\mathrm{ij}}-1 \right)D \right)^{E_{\mathrm{ij}}}}\mathrm{dz}+\int_{0}^{D} \frac{E_{\mathrm{ij}}\left( T_{\mathrm{ij}}-z-\left( E_{\mathrm{ij}}-1 \right)D \right)^{E_{\mathrm{ij}}-1}}{\left( T_{\mathrm{ij}}-\left( E_{\mathrm{ij}}-1 \right)D \right)^{E_{\mathrm{ij}}}}\mathrm{zdz}$

$=\frac{{D\left( T_{\mathrm{ij}}-E_{\mathrm{ij}}D \right)}^{E_{\mathrm{ij}}}}{\left( T_{\mathrm{ij}}-\left( E_{\mathrm{ij}}-1 \right)D \right)^{E_{\mathrm{ij}}}}+\frac{-{D\left( T_{\mathrm{ij}}-E_{\mathrm{ij}}D \right)}^{E_{\mathrm{ij}}}+\frac{1}{E_{\mathrm{ij}}+1}\left\{ \left( T_{\mathrm{ij}}-\left( E_{\mathrm{ij}}-1 \right)D \right)^{E_{\mathrm{ij}}+1}-\left( T_{\mathrm{ij}}-E_{\mathrm{ij}}D \right)^{E_{\mathrm{ij}}+1} \right\}}{\left( T_{\mathrm{ij}}-\left( E_{\mathrm{ij}}-1 \right)D \right)^{E_{\mathrm{ij}}}}$

$=\frac{\frac{1}{E_{\mathrm{ij}}+1}\left\{ \left( T_{\mathrm{ij}}-\left( E_{\mathrm{ij}}-1 \right)D \right)^{E_{\mathrm{ij}}+1}-\left( T_{\mathrm{ij}}-E_{\mathrm{ij}}D \right)^{E_{\mathrm{ij}}+1} \right\}}{\left( T_{\mathrm{ij}}-\left( E_{\mathrm{ij}}-1 \right)D \right)^{E_{\mathrm{ij}}}}$

$=\frac{T_{\mathrm{ij}}-\left( E_{\mathrm{ij}}-1 \right)D}{E_{\mathrm{ij}}+1}\left\{ 1-\left( 1-\frac{D}{T_{\mathrm{ij}}-\left( E_{\mathrm{ij}}-1 \right)D} \right)^{E_{\mathrm{ij}}+1} \right\}$ . (A.7)

### Hence equation (2) in the main text.

### For situations that $\boldsymbol{D\ll}\mathbf{T}_{\mathbf{j}}$, $\left( \mathbf{1-}\frac{\mathbf{D}}{\mathbf{T}_{\mathbf{ij}}\mathbf{-}\left( \mathbf{E}_{\mathbf{ij}}\mathbf{-1} \right)\mathbf{D}} \right)^{\mathbf{E}_{\mathbf{ij}}\boldsymbol{+1}}\boldsymbol{\approx1-}\frac{\left( \mathbf{E}_{\mathbf{ij}}\mathbf{+1} \right)\mathbf{D}}{\mathbf{T}_{\mathbf{ij}}\mathbf{-}\left( \mathbf{E}_{\mathbf{ij}}\mathbf{-1} \right)\mathbf{D}}$ according to the Taylor's theorem. It follows that $\mathbf{E}\left[ \mathbf{N.A.R}\left( \mathbf{E}_{\mathbf{ij}} \right)\boldsymbol{|}\mathbf{Z}_{\mathbf{i,1}}\boldsymbol{\geq D,\ldots,}\mathbf{Z}_{\mathbf{i,E}_{\mathbf{ij}}\mathbf{-1}}\boldsymbol{\geq D,}\mathbf{E}_{\mathbf{ij}}\boldsymbol{,}\mathbf{T}_{\mathbf{ij}} \right]\boldsymbol{\approx}\frac{\mathbf{T}_{\mathbf{ij}}\mathbf{-}\left( \mathbf{E}_{\mathbf{ij}}\mathbf{-1} \right)\mathbf{D}}{\mathbf{E}_{\mathbf{ij}}\mathbf{+1}}\left\{ \mathbf{1-}\left( \mathbf{1-}\frac{\left( \mathbf{E}_{\mathbf{ij}}\mathbf{+1} \right)\mathbf{D}}{\mathbf{T}_{\mathbf{ij}}\mathbf{-}\left( \mathbf{E}_{\mathbf{ij}}\mathbf{-1} \right)\mathbf{D}} \right) \right\}\mathbf{=D}$. Under such circumstances, equation (A.1) becomes

###

$$\mathbf{E[T}_{\mathbf{ij}}\mathbf{-}\mathbf{T}_{\mathbf{ij}}^{\mathbf{*}} \boldsymbol{|}\mathbf{Z}_{\mathbf{i,1}}\boldsymbol{\geq D,\ldots,}\mathbf{Z}_{\mathbf{i,E}_{\mathbf{ij}}\mathbf{-1}}\boldsymbol{\geq D,}\mathbf{E}_{\mathbf{ij}}\boldsymbol{,}\mathbf{T}_{\mathbf{ij}}\mathbf{]}\boldsymbol{\approx}\left( \mathbf{E}_{\mathbf{ij}}\mathbf{-1} \right)\boldsymbol{\times D+D}\boldsymbol{=}\mathbf{E}_{\mathbf{ij}}\boldsymbol{\times D}$$

which implies that on the group level, with a sufficiently large sample size for each group, $T_{j}^{*}\approx T_{j}-E_{j}\times D$ , hence equation (1) in the main text.
